# Gene Therapy and Chronic Pain

**DOI:** 10.1100/tsw.2006.197

**Published:** 2006-08-31

**Authors:** Doris K. Cope, William R. Lariviere

**Affiliations:** Department of Anesthesiology, University of Pittsburgh School of Medicine, Pittsburgh, PA, USA

**Keywords:** gene therapy, genetic vectors, enkephalins, growth factors, interleukins

## Abstract

Chronic, unremitting pain is perhaps the most common reason that patients seek medical care. In general, conservative techniques, such as medical management, are implemented as first-line therapy. Local anesthesia and lytic procedures, followed by interventional techniques, such as dorsal column stimulation and intrathecal drug delivery systems, are second-line therapies. However, for refractory and severe pain, which is not adequately controlled by other modes of therapy, new emerging options, including molecular or gene therapy, may become more widely utilized as experimental results are translated into clinical options.

## THE NEED FOR ALTERNATE PAIN THERAPIES

Chronic, unremitting pain is perhaps the most common reason that patients seek medical care. Chronic pain is particularly present in advanced cancer and in chronic diseases, such as autoimmune and neuropathic pain syndromes[1,2]. Of patients with advanced cancer, over half report severe pain. In many cases, adequate pain relief is not achieved by current modes of therapy and is limited by unacceptable side effects. For patients who fail interventional therapies or intrathecally administered drugs, gene therapy may promise improved pain control. Currently, gene therapy is primarily experimental, with different vectors and techniques being employed. A brief review of the field, followed by the limited human data to date, will be discussed.

## METHODS OF GENE THERAPY

Molecular or gene therapy introduces genetic material for the overexpression of endogenous analgesic compounds or anti-inflammatory compounds, or to inhibit genetic transcription of nociceptive compounds[3,4,5,6]. One goal is to decrease excitability of spinal cord neurons that receive input from primary afferent fibers that signal injury or pathology in the affected tissue, or to modulate the function of activated glial cells that contribute to pain processing in the central nervous system. Animal studies have not demonstrated significant adverse findings that would signal caution. However, large-scale clinical trials in human patients remain to be performed to assess short- and long-term risk.

Several experimental methods are currently available for the introduction of transgenic material to the target tissue[4,7,8]. Each method uses a different vector for introduction and transduction of the transgene, and has been associated with different levels of immunogenicity, toxicity, target cell selectivity, efficiency of gene transduction, and transgene insert capability. Certain properties are more suited to the treatment of prolonged pain than others. A discussion of the major treatment types follows.

## Liposomes

Liposomes are positively charged lipid membranes that complex with DNA and fuse with negatively charged cell membranes, which results in the transfer of DNA into the cells. Since liposomes are not target specific, a wide variety of liposomes are available for gene transfer to a wide range of cell targets. An advantage is the lower immune response that is induced by liposomes. However, this method also results in low gene transduction efficiency. Therefore, this vector is less promising as a mechanism for inducing pain relief in patients.

## Naked Plasmid DNA

The use of naked plasmid double stranded (ds) DNA without viral vector components, like liposomes, is also without immunological response to the vector components and, therefore, there is less concern about related damage to host DNA as compared to viral vector systems. For the control of pain in animal models, gene gun injection of naked plasmid DNA has proven effective[9,10].

One example is to apply gene therapy via naked plasma DNA, encoding in vascularly modified endothelial growth factor-2 (VEGF-2), which increases myocardial blood flow, reducing myocardial ischemia and angina. However, in an early clinical application, one patient died after VEGF-2 DNA was injected via a thoracotomy into his ischemic myocardium. In a later study conducted by this same group, Dr. Richard A. Schatz and associates demonstrated significant reduction in pain and angina class for 2 years post-treatment without significant patient morbidity. A large Phase III outcome trial is underway[11].

## Viral Vectors

Viral models for *in vivo* gene transfer for pain reduction in animal models are more commonly utilized than liposomes or naked plasma DNA. These various viral vectors include adenovirus, adeno-associated virus, retrovirus, and herpes simplex virus vectors.

The most promising is the adenovirus vector, which shows a high efficiency for the infection of both dividing and nondividing cells, unlike single-stranded RNA retrovirus vectors that require dividing cells. Normal adenoviral infection targets are intestinal epithelial cells, although in subpopulations of elderly and children, respiratory epithelium may be affected[12]. Some target cell specificity is demonstrated by infection of predominantly meningeal cells after lumbosacral intrathecal injection of adenovirus vectors[13].

A major limitation of adenoviral vectors is that the high turnover of target cells (gut epithelium) leads to transient expression of only days to weeks[14]. Thus, multiple applications would be required to treat a chronic condition. In addition, adenovirus vectors induce proinflammatory factors that may increase pain signaling[15,16]. A modification of the adenovirus vector, adeno-associated virus (AAV) vectors, harmless by themselves, also do not stimulate inflammation or antibody production, but can be associated with persistent transgene expression. With AAV infection of the sciatic nerve, for instance, small, medium, and large fiber types of the dorsal root ganglion have been shown to be effectively targeted[17].

## Herpes Simplex Virus (HSV)-1

Most intriguing and of greatest potential for somatic chronic pain therapy is HSV vector–mediated gene therapy[18]. Primary afferent neurons are the natural targets of HSV, conferring an advantage over other vector systems for which the target cells are not well characterized. After subcutaneous injection or even topical application, the double-strand DNA HSV vector infects epithelial cells or neurons directly, undergoes several cycles of replication, penetrates peripheral afferent nerve terminals, and is retrogradely transported to cell bodies in dorsal root sensory ganglia. It remains there as chromatin structure without integration into the host genome. This virus can also carry three to four times the length of DNA than can be inserted in the adenovirus vector. The target cell specificity, the possibility of introducing multiple synergistic transgenes, the ease of administration, and limited side effects make the HSV vector particularly well suited for gene therapy in chronic pain.

## PRECLINICAL STUDIES OF GENE THERAPY FOR PROLONGED PAIN

Transgenes have been delivered by gene vector systems to reduce nociception in animals by the overexpression of genes whose products are analgesic. This includes genes for enkephalins to promote analgesia, as well as the inhibition of genes whose products are pain producing, such as calcitonin gene-related peptide (CGRP)[5,6,19]. In addition, non-neuronal cells are affected, such as astrocytes and microglia known to modulate spinal cord neuron activity by the introduction of interleukin-10 (IL-10) genes whose products act on glial cell receptors not found on spinal cord neurons (see Tables 1–3)[20,21].

**TABLE 1**
**Effect of Gene Therapy on Baseline Thermal Pain and Mechanical Sensitivity in Rodents**

| Study | Vector System | Transgene | Pain Model | Results |
|---|---|---|---|---|
| Finegold et al. 1999 | Adenovirus, intrathecal | β-Endorphin | Thermal pain | No effect |
| Gu et al. 2005 | AAV, sciatic nerve | μ-Opioid receptor | Thermal pain | No effect |
| Lu et al. 2002 | Gene gun, plasmid DNA, cutaneous | Proopiomelanocortin | Thermal pain | No effect |
| Milligan et al. 2005a,b | Adenovirus, intrathecal | Interleukin-10 | Thermal pain, mechanical sensitivity | No effect |
| Tan et al. 2005 | Naked RNA, intrathecal | NMDA receptor NR2B subunit siRNA | Thermal pain | No effect |
| Wilson et al. 1999 | HSV, cutaneous | Proenkephalin A | Thermal pain | No effect |
| Yao et al. 2003 | Adenovirus, intrathecal | IL-2 | Thermal pain | Analgesia |

Ideally, pain should be selectively affected with normal sensation spared since effects on basal thermal or mechanical sensitivity have not been demonstrated in studies to date. Almost all of the recent studies have shown that gene therapy, while exhibiting antinociceptive, antihyperalgesic, or antiallodynic effects, does not affect the basal sensitivity to von Frey monofilaments or to radiant heat focused on the hind paw of the rat (see Table 1)[17,20,21,22,23,24,25,26]. This finding is true regardless of the transgene, gene delivery system, and mode and site of administration (see Table 1). Only one exception without any clear explanation has been described: adenovirus vector delivery of IL-2 produced significant thermal antinociception for up to 3 weeks[26].

**TABLE 2**
**Effect of Gene Therapy on Inflammatory Pain Sensitivity in Rodents**

| Study | Vector System | Transgene | Pain Model | Results |
|---|---|---|---|---|
| Braz et al. 2001 | HSV, SC | Proenkephalin A | Polyarthritic thermal hyperalgesia | Analgesia |
| Chuang et al. 2005 | Gene gun, plasmid DNA, bladder | Preproenkephalin | Inflammatory bladder irritation | Analgesia |
| Finegold et al. 1999 | Adenovirus, intrathecal | β-Endorphin | Inflammatory thermal hyperalgesia | Analgesia |
| Garry et al. 2000 | Naked DNA, intrathecal | NMDA-R1 antisense oligonucleotides | Acute inflammatory pain | Analgesia |
| Goss et al. 2001 | HSV, cutaneous | Proenkephalin | Acute inflammatory pain | Analgesia |
| Gu et al. 2005 | AAV, sciatic nerve | μ-Opioid receptor | Monoarthritic thermal hyperalgesia | No effect |
| Kang et al. 1998 | HSV, amygdala | Proenkephalin | Acute inflammatory pain | Analgesia |
| Lu et al. 2002 | Gene gun, plasmid DNA, cutaneous | Proopiomelanocortin | Acute inflammatory pain | Analgesia |
| Milligan et al. 2005a,b | Adenovirus, Intrathecal | IL-10 | Inflammatory mechanical allodynia | Analgesia |
| Tan et al. 2005 | Naked RNA, intrathecal | NMDA receptor NR2B subunit siRNA | Acute inflammatory pain | Analgesia |
| Wilson et al. 1999 | HSV, cutaneous | Proenkephalin A | Inflammatory thermal hyperalgesia | Analgesia |
| Wilson and Yeomans 2002 | HSV, cutaneous | CGRP antisense | Acute inflammatory pain | Analgesia |
| Yao et al. 2002 | Plasmid DNA, intrathecal, SC | IL-2 | Inflammatory thermal hyperalgesia | Analgesia |

## Inflammatory Pain

Numerous studies to date have demonstrated the effectiveness of gene therapy in limiting inflammatory nociception in the rat or mouse (see Table 2). In the commonly used formalin inflammatory pain test, a dilute solution of formaldehyde is injected subcutaneously in the hind paw, evoking a biphasic response of spontaneous nociceptive behaviors of paw licking, lifting, and shaking[27]. Other diverse models of inflammatory hypersensitivity, which differ genetically from the formalin-induced pain model, have demonstrated inhibition of capsaicin or DMSO-induced thermal hyperalgesia by HSV vector delivery of enkephalins[10,27,28,29]. HSV delivery of CGRP antisense released from lumbar primary sensory neurons decreased inflammatory hyperalgesia and decreased swelling in an animal model of rheumatoid arthritis[4,30]. In addition, cytokine-directed gene therapy results in an analgesic effect[20,21,22,31]. Duration of effect has been demonstrated to range from 7 days to 14 weeks. Low back pain is being evaluated in animal models of degenerative disc disease to block the inflammatory response from herniated discs and regenerative diseased discs. Genetic therapies are being evaluated with the intent of developing human therapies for similar pathology[32].

## Neuropathic Pain

Neuropathic pain differs from somatic and visceral pain in clinical presentation, mechanism, and treatments. Several recent studies have demonstrated effectiveness of gene therapy in models of neuropathic pain (see

**TABLE 3**
**Effect of Gene Therapy on Neuropathic Pain Models in Rodents**

| Study | Vector System | Transgene | Pain Model | Results |
|---|---|---|---|---|
| Eaton et al. 2002 | AAV, spinal cord | Brain-derived neurotrophic factor | Sciatic nerve constriction, thermal and mechanical hyperalgesia | Analgesia |
| Hao et al. 2003 | HSV, SC | Glial cell-derived neurotrophic factor | Lumbar spinal nerve ligation, mechanical allodynia | Analgesia |
| Hao et al. 2003 | HSV, SC | Proenkephalin | Lumbar spinal nerve ligation, mechanical allodynia | Analgesia |
| Hao et al. 2005 | HSV, SC | Glutamic acid decarboxylase | Lumbar spinal nerve ligation, thermal and mechanical hyperalgesia | Analgesia |
| Lin et al. 2002 | Naked DNA, intrathecal | Proopiomelanocortin | Sciatic nerve constriction, thermal hyperalgesia | Analgesia |
| Liu et al. 2004 | HSV, SC | Glutamic acid decarboxylase | Spinal cord hemisection, mechanical allodynia | Analgesia |
| Meunier et al. 2005 | HSV, vibrissal area | Preproenkephalin A | Infraorbital nerve constriction, mechanical allodynia | Analgesia |
| Milligan et al. 2005a,b | Adenovirus, intrathecal | IL-10 | Sciatic nerve constriction, thermal and mechanical hyperalgesia | Analgesia |
| Pradat et al. 2001 | Adenovirus, muscle | Neurotrophin-3 | Streptozotocin diabetic peripheral neuropathy, acrylamide intoxication | Analgesia |
| Yao et al. 2002 | Plasmid DNA, intrathecal | IL-2 | Sciatic nerve constriction, thermal hyperalgesia | Analgesia |
| Yao et al. 2003 | Adenovirus, intrathecal | IL-2 | Sciatic nerve constriction, thermal hyperalgesia | Analgesia |
| Yeomans et al. 2004 | HSV, cutaneous | Preproenkephalin | Intrathecal pertussis toxin, thermal hyperalgesia | Analgesia |

Table 3)[33]. Animal models include ligation of the fifth lumbar nerve distal to the dorsal root ganglion, chronic constriction injury (CCI) of the sciatic nerve or infraorbital nerve, intrathecal pertussis toxin injection[20,21,26,34,35,36,37]. Gene therapy has increased expression of various neurotrophic factors and capitalized on the role of GABA by increasing glutamic acid decarboxylase, which attenuated pain in neuropathic pain models[38,39,40,41,42,43,44,45. Effectiveness ranged from less than a week to 8 weeks, with reinstatement of effects with reinoculation[41,42,45]. GABAergic therapy for neuropathic pain has had limited success, but gene transfer of glutamic acid decarboxylase into the dorsal root ganglion of rats via an HSV basal vector injected under the skin showed promise in treating this refractory type of pain[46]. High titers of missing gene products can be obtained from rodent models of brain tumor, Parkinsonism, and spinal root and spinal cord injury via nonreplicating HSV vectors[46]. Most importantly, these vectors can be produced in pure preparations to high titers and, thus, it would be feasible to begin testing in human patients. However, human data are currently lacking.

### Cancer Pain

One published study has examined the effects of gene therapy in a rat model of cancer pain. Goss et al. have shown that inoculation with the pEnkephalinA-encoding HSV vector produces significant antinociception mediated by opioid receptors in the spinal cord in a mouse femoral bone cancer pain model[47]. Presently gene therapy is moving from the laboratory to Phase I/II clinical trials primarily in the area of localized cancer pain.

Clinical trials with isolated vertebral metastases are also in the planning stage. In contrast to the limited cell targets of HSV vectors, intrathecal delivery of AAV vectors can potentially deliver the transgene to multiple sites in the spinal canal[48]. An early study intraprostatically injected adenovirus in 16 patients, delivering suicide genes to sensitize malignant cells to radiation and chemotherapy. There were no treatment-related serious adverse effects. Over half of the patients demonstrated a positive response of varying degrees[49].

## CURRENT STATUS OF HUMAN CLINICAL TRIALS

According to the *Journal of Gene Medicine* at the end of March 2006, there were 1,145 active clinical gene therapy trials with 2.1% in Phase III (n = 24). The vast majority were cancer therapies (n = 762; 66.6%). Since gene therapy is novel, without long-term human outcome data, FDA approval is more readily granted for patients with life-limiting disease. A combination of monogenetic, vascular, and infectious diseases (n = 275) comprised 24% of all clinical trials. The majority of these studies are being conducted in North America (67%) and Europe (29%). The most common vectors currently being used in these clinical trials are adenovirus and AAV vectors (28.4%) followed by lipofection (8.3%) and HSV vectors (3.3%). Other vectors being trialed in human studies comprise less than 0.5% of current studies, including the following vectors: adenovirus + retrovirus, flavivirus; gene gun, lentivirus, *Listeria monocytogenes*, and measles virus[50].

Since the vast majority of human trials have been to modify disease states, trials for the treatment of chronic pain have been sparse. One interesting approach is identifying combinations of specific genetic mutations in a particular disease state, such as an autoinflammatory disorder, and then tailoring therapy appropriately. An example is classifying autoimmune disorders such as Crohn’s disease and hereditary periodic fevers by constellations of causation genes rather than based on physiological symptoms that frequently overlap. Pain is also a generalized response to many different syndromes, and type of pain as well as underlying disorders vary widely. Complex genetic screening cannot only help to diagnose a disorder, but also to direct gene therapy to treat the specific mutations rationally. In this same manner, various pain syndromes may, in the future, be genotyped for specificity in both diagnosis and treatment.

## CONCLUSIONS AND FUTURE DIRECTIONS

The significant effects of gene therapy on pain can occur with or without significant changes in degree of tissue injury or pathology. In the treatment of chronic pain, it is necessary to address both acute insult as well as chronic changes resulting from persistent pain. Therapeutic options including the use of vector systems, and especially of the HSV vectors in which several transgenes can be inserted to act synergistically, promise to be a fruitful future direction of the research. Methods to improve the vector systems remain under investigation, including modifications to enhance target cell selectivity, reduce evoked inflammatory and toxic responses, and increase the efficiency and duration of transgene expression[7,51]. Transduction promoters are also actively being varied to increase expression while minimizing toxicity[30].

This emerging technology has great potential for widespread clinical applications that are supported by preclinical studies. The available results from animal models of gene therapy for prolonged pain demonstrate gene therapy to be clearly effective, independent of the etiological mechanisms of pain and regardless of whether specific nerves such as the trigeminal nerve, or whether somatic or visceral pain systems are targeted. Still, to date, the indications and contraindications are not yet determined and will need to be well understood before widespread clinical application is adopted.
